# Use of sepsis-related diagnostic criteria in primary care: a survey among general practitioners

**DOI:** 10.1093/fampra/cmab020

**Published:** 2021-03-23

**Authors:** Merijn C F Mulders, Feike J Loots, Joey van Nieuwenhoven, Jan C ter Maaten, Hjalmar R Bouma

**Affiliations:** 1 Department of Internal Medicine, University of Groningen, University Medical Center Groningen, Groningen, The Netherlands; 2 Julius Center for Health Sciences and Primary Care, University Medical Centre Utrecht, Utrecht, The Netherlands; 3 Het College, Weert, The Netherlands; 4 Department of Clinical Pharmacy and Pharmacology, University of Groningen, University Medical Center Groningen, Groningen, The Netherlands

**Keywords:** Early warning score, general practitioners, infectious disease medicine, organ dysfunction scores, primary health care, sepsis

## Abstract

**Background:**

Use of sepsis-criteria in hospital settings is effective in realizing early recognition, adequate treatment and reduction of sepsis-associated morbidity and mortality. Whether general practitioners (GPs) use these diagnostic criteria is unknown.

**Objective:**

To gauge the knowledge and use of various diagnostic criteria. To determine which parameters GPs associate with an increased likelihood of sepsis.

**Methods:**

Two thousand five hundred and sixty GPs were invited and 229 agreed to participate in a survey, reached out to through e-mail and WhatsApp groups. The survey consisted of two parts: the first part aimed to obtain information about the GP, training and knowledge about sepsis recognition, and the second part tested specific knowledge using six realistic cases.

**Results:**

Two hundred and six questionnaires, representing a response rate of 8.1%, were eligible for analysis. Gut feeling (98.1%) was the most used diagnostic method, while systemic inflammatory response syndrome (37.9%), quick Sequential Organ Failure Assessment (qSOFA) (7.8%) and UK Sepsis Trust criteria (UKSTc) (1.5%) were used by the minority of the GPs. Few of the responding GPs had heard of either the qSOFA (27.7%) or the UKSTc (11.7%). Recognition of sepsis varied greatly between GPs. GPs most strongly associated the individual signs of the qSOFA (mental status, systolic blood pressure, capillary refill time and respiratory rate) with diagnosing sepsis in the test cases.

**Conclusions:**

GPs mostly use gut feeling to diagnose sepsis and are frequently not familiar with the ‘sepsis-criteria’ used in hospital settings, although clinical reasoning was mostly in line with the qSOFA score. In order to improve sepsis recognition in primary care, GPs should be educated in the use of available screening tools.

**Key Messages**

- Recognition of sepsis remains a major health care challenge.
- Research has shown that use of screening tools can improve sepsis recognition.
- In this study, GPs mostly reported to use gut feeling to diagnose sepsis.
- Most GP did not use sepsis-criteria in the diagnosis of sepsis.
- The qSOFA proved to be most in line with the clinical reasoning of GPs.
- Thus, educating GPs in the use of screening tools can improve sepsis recognition.

## Introduction

Sepsis is defined as a dysregulated and disproportionate inflammatory response to an infection, causing life-threatening organ failure (1) and annually affects around 49 million people worldwide (2). In the Netherlands, this results in around 13 000 hospital admissions per year (3). Sepsis is the leading cause of death worldwide with an in-hospital mortality rate of 21%, resulting in an estimate of 11 million deaths per year (2,4,5). Reducing the morbidity and mortality associated with sepsis remains a major health care challenge (6). Over the past decade, substantial progress has been made in hospital settings, especially with the launch of the Surviving Sepsis Campaign (SSC) in 2004 (7,8). Early recognition and initiation of adequate treatment are crucial for successful treatment of a patient with sepsis (9). Measurement of vital signs is part of triage as performed in patients presenting to the emergency department (ED), while abnormal vital signs included in ‘sepsis-criteria’ should trigger treatment guided by a sepsis protocol (10): a key step that is critical for the success of the SSC (7,8). Several tools support the assessment of a potentially septic patient in hospitals, of which the most well known are the systemic inflammatory response syndrome (SIRS) criteria and the quick Sequential Organ Failure Assessment (qSOFA) score (11). The current consensus definitions advise the use of the qSOFA, a clinical decision tool for bedside evaluation, to identify patients with sepsis out-of-hospital, in the ED and on general wards (1). The qSOFA consists of an altered mental status, a respiratory rate of ≥22/minute and a systolic blood pressure <100 mmHg. The qSOFA is positive when at least two of these items are present and implies an increased risk of sepsis-related mortality (1).

Recognition of sepsis in pre-hospital settings is poor and is associated with increased mortality (12–14). Most patients with sepsis initially contact a general practitioner (GP), assessment by their GP is therefore crucial for early detection and timely initiation of treatment (14,15). In the UK, GPs are encouraged by the National Institute for Health and Care Excellence (NICE) to obtain a full set of vital signs in order to efficiently risk-stratify patients with a suspected sepsis (16,17). Use of screening tools, based on the SSC diagnostic criteria, can improve sepsis recognition in pre-hospital settings (12,18). In addition to the qSOFA criteria, the UK Sepsis Trust has developed a screening and action tool that accurately reflects the recommendations in the NICE guidelines (19). However, whether GPs actually use diagnostic criteria to recognize sepsis is unknown. The objective of this study was to assess whether aforementioned sepsis-criteria, such as the SIRS, qSOFA and UK Sepsis Trust, are known to and used by GPs, and which signs GPs associate with an increased likelihood of sepsis.

## Methods

### Design, procedure and participants

An online cross-sectional survey (Supplementary Figure S1) was dispersed among GPs in the Netherlands in September 2019. Study data were collected and managed using REDCap electronic data capture tools hosted at the University Medical Center Groningen (20,21). REDCap (Research Electronic Data Capture) is a secure, web-based software platform designed to support data capture for research studies. The Strengthening the Reporting of Observational Studies in Epidemiology (STROBE) guidelines, available at the Enhancing the QUAlity and Transparency Of health Research (EQUATOR) website, were used to ensure the reporting of this cross-sectional study (22). A reminder was sent 2 weeks after the initial date, while data collection ended after 8 weeks. Unanswered questions were regarded as missing data in the analyses. Surveys were excluded from the analysis if none of the cases were completed. GPs were addressed using mailing lists, a digital newsletter and a nation-wide WhatsApp group chat provided by three GP research groups. First, a random sample of about 1300 e-mail addresses was taken from a digital address list that contained e-mail addresses of all GPs in the Netherlands. Next, a digital newsletter was sent to all GPs subscribed to ‘Coöperatie Huisarts Utrecht en Stad’, a general GP newsletter, reaching about 160 GPs. Finally, research groups located in Groningen, Nijmegen and Utrecht reached out to 1100 GPs with either academic as well as teaching practices via WhatsApp. In total, 2560 GPs were asked to participate in the survey.

### Survey

The survey was developed by the research team with input of internists specialized in acute medicine, emergency physicians and GPs focussed on research in General Practice Medicine. The survey focussed on parameters that add to the diagnosis of sepsis and consisted of two sections. The first part contained questions about the individual GP, their practice, the amount of time spent in out-of-hours primary care and their familiarity with sepsis-related diagnostic criteria. The second part comprised of six fictitious cases of patients with suspected sepsis a GP can encounter in daily practice. A summary of the anamnesis of about 5–10 sentences and the performed physical examination, consisting of general appearance and vital parameters, was given (Supplementary Table S1). The GP had to determine how likely sepsis was, which parameters add to the diagnosis and what treatment should be issued. Cases were defined as true sepsis by an expert panel. The expert panel consisted of 10 acute internal medicine specialists. All panel members independently assessed the likelihood of sepsis in the six fictitious cases. The majority opinion of the expert panel was used for the classification of the cases. A ‘correct’ sepsis diagnosis was defined as marking a true sepsis case as sepsis is likely or very likely.

### Data analysis

The Shapiro–Wilk test was used to test for normality. For normally distributed data, the mean and standard deviation were calculated. For non-normally distributed data, the median and interquartile ranges were calculated. For categorical variables, frequency and percentage of cases were calculated. Categorical data were analysed with the chi-square test.

A multilevel binomial logistic regression analysis was used to identify parameters associated with sepsis. Random effects were used to correct for the clustering effect on the level of the responding GPs. Two separate analyses were performed for the outcomes ‘sepsis according to the GP’ (either ‘sepsis is likely’ or ‘sepsis is very likely’) and ‘correct diagnosis of sepsis’. Parameters that only occurred in some of the cases, like diuresis, recent chemotherapy and rash, were given the value of 0, or neutral, when missing. In total, 19 parameters were used for the analyses, including both GP characteristics as well as symptoms and signs from the cases.

All statistical analyses were performed using IBM SPSS Statistics for Windows, version 23.0. A *P* value of ≤0.05 was considered significant; all tests were two tailed.

## Results

### Participating GPs

Two hundred and twenty-nine GPs (8.9%), out of the 2560 GPs that were reached out to, responded to our survey. Of these, 23 (10%) had to be excluded as none of the six fictitious cases were completed. A non-response analysis showed that the excluded responses did not differ from the total sample in age, sex and years of experience. Given a total number of GPs in the Netherlands of around 12 000 (23), a completed survey of 2% of the total Dutch GP population was received. 54.9% of the responders were female, with a median age of 49.5 and median of 16 years of experience. Both responders from (very) strongly urban areas (29.6%) and acting GPs (16.5%) were underrepresented in our population (23). The minority of the responding GPs received sepsis training in the past 5 years (20.9%) or were trained as a GP specialized in emergency care (2.4%) (Table 1).

**Table 1.** Comparison of the main characteristics of the 206 responding Dutch GPs with the available data of the source population of 12 446 GPs in the Netherlands (2019)

Background characteristics	*n* = 206	*n*=12 446
Age [median (IQR)]	49.5 (41.0; 58.0)	48
Female [*n* (%)]	113 (54.9)	6692 (53.8)
Years of experience [median (IQR)]	16 (7.5; 22.5)	
Working area [*n* (%)]		
*Very strongly urban**	26 (12.6)	2962 (23.8)
*Strongly urban**	35 (17.0)	3672 (29.5)
*Moderately urban***	52 (25.2)	2004 (16.1)
Little urban	46 (22.3)	2763 (22.2)
*Non-urban****	47 (22.8)	1045 (8.4)
Working as acting GP [*n* (%)]	34 (16.5)	2348 (18.9)
Size of practice^a^ [*n* (%)]		
1000–2000	37 (18.0)	
2000–3000	91 (44.2)	
>3000	44 (21.4)	
Number of monthly out-of-hours duties [*n* (%)]		
0–1	37 (18.0)	
2–4	151 (73.2)	
≥5	18 (8.7)	
Sepsis training in the past 5 years [*n* (%)]	43 (20.9)	
Specialized in acute medicine^b^ [*n* (%)]	5 (2.4)	

IQR, interquartile range.

^a^In number of patients.

^b^Registered as ‘NHG-kaderarts Huisarts en Spoedzorg’.

*Significant difference between sample and source population: **P < 0.005, ***P < 0.05, ****P < 0.001.

### Diagnostic methods used to diagnose sepsis

GPs most frequently reported to use gut feeling to recognize sepsis (98.1%). Ongoing deterioration and an abnormal presentation of the individual patient were deemed important when recognizing a potential septic patient. Even though most GPs had heard of SIRS (75.7%), the minority of the responding GPs was familiar with qSOFA (27.7%) or UK Sepsis Trust criteria (UKSTc) (11.7%) (Table 2). Only the minority of the GPs used SIRS, qSOFA or UKSTc to aid sepsis recognition (respectively, 37.9%, 7.8% and 1.5%) (Table 3).

**Table 2.** Familiarity of the 206 responding Dutch GPs with various diagnostic criteria (2019)

Diagnostic criteria	*n* = 206
Familiar with SIRS [%, (95% CI)]	
Yes, well known	22.3 (16.8; 28.6)
Yes, heard of them	53.4 (46.3; 60.4)
No, never heard of	24.3 (18.6; 30.7)
Familiar with qSOFA [%, (95% CI)]	
Yes, well known	6.3 (3.4; 10.6)
Yes, heard of them	21.4 (16; 27.6)
No, never heard of	72.3 (65.7; 78.3)
Familiar with UK Sepsis Trust rule [%, (95% CI)]	
Yes, well known	1.5 (0.3; 4.2)
Yes, heard of them	10.2 (6.4; 15.2)
No, never heard of	88.3 (83.2; 92.4)
Not familiar with SIRS, qSOFA or UK Sepsis Trust rules [%, (95% CI)]	22.8 (17.3; 29.2)

CI, confidence interval.

**Table 3.** Diagnostic methods used by the 206 responding Dutch GPs (2019)

Diagnostic method used	*n* = 206 (95% CI)
Gut feeling [%, (95% CI)]	98.1 (95.1; 99.5)
SIRS [%, (95% CI)]	37.9 (31.2; 44.9)
qSOFA [%, (95% CI)]	7.8 (4.5; 12.3)
UK Sepsis Trust [%, (95% CI)]	1.5 (0.3; 4.2)
Anxious family [%, (95% CI)]	36.9 (30.3; 43.9)
Ongoing deterioration [%, (95% CI)]	74.8 (68.3; 80.5)
Abnormal presentation [%, (95% CI)]	58.3 (51.2; 65.1)

CI, confidence interval.

### Variation in diagnosing sepsis

When diagnosing sepsis in the fictitious cases, answers ranged from sepsis is (very) unlikely to sepsis is (very) likely in every case (Table 4).

**Table 4.** Likelihood of sepsis according to the 206 responding Dutch GPs (2019)

Cases [*n* (%)]	Sepsis is very unlikely	Sepsis is unlikely	Sepsis is possible	Sepsis is likely	Sepsis is very likely	Total
Case 1^a^	2 (1)	0 (0)	6 (2.9)	34 (16.5)	164 (79.6)	206 (100)
Case 2	4 (1.9)	31 (15.7)	131 (66.5)	26 (13.2)	5 (2.4)	197 (95.6)
Case 3^a^	1 (0.6)	0 (0)	44 (24.4)	82 (45.6)	53 (29.4)	180 (87.4)
Case 4	0 (0)	20 (11.4)	95 (54.3)	45 (25.7)	15 (8.6)	175 (85)
Case 5^a^	0 (0)	1 (0.6)	21 (12.2)	64 (37.2)	86 (50)	172 (83.5)
Case 6	1 (0.6)	15 (8.8)	75 (44.1)	60 (35.3)	19 (11.2)	170 (82.5)

^a^True sepsis according to the expert panel.

### Identification of factors associated with diagnosing sepsis

GPs who received sepsis training in the past 5 years were more likely to mark a case as likely or very likely sepsis (*P* = 0.002). Parameters GPs most strongly associated with an increased likelihood of sepsis as diagnosis were respiratory frequency, blood pressure, capillary refill time and mental status (*P* < 0.001). Clinical impression (*P* = 0.003), oxygen saturation (*P* = 0.021), recent chemotherapy (*P* = 0.005) and the presence of a rash (*P* = 0.037) were other parameters GPs valued when diagnosing sepsis (Table 5).

**Table 5.** Results of a logistic regression model estimating the association between individual parameters and, respectively, a GP diagnosis of sepsis and a ‘correct’ sepsis diagnosis (2019)

Variables in the equation	GP diagnosis of sepsis			Correct (adjudicated) diagnosis of sepsis		
	RC (SE)	OR (95% CI)	*P* value	RC (SE)	OR (95% CI)	*P* value
GP characteristics						
Female	−0.059 (0.219)	0.942 (0.613; 1.449)	0.786	0.008 (0.196)	1.008 (0.686; 1.481)	0.968
In-service sepsis education^a^	0.881 (0.288)	2.414 (1.372; 4.247)	**0.002**	−0.205 (0.243)	0.815 (0.506; 1.312)	0.399
>10 years of experience	−0.227 (0.246)	0.797 (0.491; 1.292)	0.357	0.152 (0.215)	1.164 (0.764; 1.773)	0.480
≥2 monthly out of hours duties	−0.525 (0.291)	0.591 (0.334; 1.046)	0.071	0.396 (0.245)	1.486 (0.919; 2.401)	0.106
Use of ABCDE	0.163 (0.250)	1.178 (0.721; 1.924)	0.513	−0.041 (0.218)	0.960 (0.625; 1.473)	0.850
Familiar with the qSOFA	−0.149 (0.257)	0.862 (0.521; 1.426)	0.562	0.061 (0.227)	1.063 (0.681; 1.660)	0.562
Familiar with the UKSTc	0.106 (0.371)	1.112 (0.537; 2.300)	0.775	0.046 (0.313)	1.047 (0.567; 1.933)	0.883
Familiar with the SIRS	0.225 (0.268)	1.252 (0.740; 2.118)	0.402	−0.260 (0.244)	0.771 (0.478; 1.245)	0.287
Physical examination						
Clinical impression	0.677 (0.228)	1.968 (1.259; 3.076)	**0.003**	−0.490 (0.218)	0.613 (0.399; 0.940)	**0.025**
Oxygen saturation	0.440 (0.213)	1.553 (1.022; 2.360)	**0.021**	−0.287 (0.201)	0.750 (0.506; 1.113)	0.153
Respiratory frequency	0.957 (0.221)	2.605 (1.690; 4.015)	**<0.001**	0.417 (0.213)	1.518 (1.00; 2.303)	**0.050**
Blood pressure	1.021 (0.222)	2.776 (1.797; 4.289)	**<0.001**	−0.265 (0.213)	0.767 (0.505; 1.166)	0.214
Pulse	0.367 (0.262)	1.443 (0.863; 2.412)	0.162	−0.489 (0.259)	0.613 (0.369; 1.019)	0.059
Capillary refill time	1.045 (0.298)	2.843 (1.583; 5.104)	**<0.001**	0.842 (0.287)	2.321 (1.322; 4.076)	**0.003**
Mental status	1.470 (0.291)	4.349 (2.458; 7.696)	**<0.001**	1.324 (0.276)	3.758 (2.188; 6.455)	**<0.001**
Temperature	0.360 (0.237)	1.433 (0.901; 2.279)	0.129	−0.494 (0.227)	0.610 (0.390; 0.953)	**0.030**
Diuresis	0.135 (0.236)	1.144 (0.721; 1.816)	0.567	0.073 (0.216)	1.076 (0.704; 1.643)	0.736
Recent chemotherapy	0.695 (0.248)	2.004 (1.231; 3.261)	**0.005**	−0.838 (0.229)	0.432 (0.276; 0.678)	**<0.001**
Rash	0.939 (0.451)	2.558 (1.056; 6.196)	**0.037**	1.095 (0.429)	2.991 (1.289; 6.936)	**0.011**

95% CI, 95% confidence interval; OR, odds ratio; RC, regression coefficient; SE, standard error. Bold printed *P* values are ≤0.05.

^a^In the past 5 years.

### Identification of factors associated with a ‘correct’ sepsis diagnosis

Remarkably, in-service sepsis education, being experienced, a high frequency of out of hours duties, use of the ABCDE and being familiar with the SIRS, qSOFA or UKSTc, did not increase the likelihood of ‘correctly’ identifying cases as sepsis. Moreover, marking clinical impression (*P* = 0.025), temperature (*P* = 0.030) and recent chemotherapy (*P* < 0.001) as abnormal decreased the chance of a correct ‘sepsis’ diagnosis. Respiratory frequency (*P* = 0.050), capillary refill time (*P* = 0.003), mental status (*P* < 0.001) and the presence of a rash (*P* = 0.011) being marked as abnormal did increase the chance of a ‘correct’ sepsis diagnosis (Table 5).

## Discussion

### Summary

In this cross-sectional survey, parameters most important to a GP when diagnosing sepsis were identified based on their characteristics and realistic cases. With a response rate of 8.9%, 206 GPs, with a total of 1089 completed fictitious cases, were selected for the analysis. Gut feeling, ongoing deterioration and abnormal presentation were the most used diagnostic methods for sepsis, diagnostic criteria like the SIRS, qSOFA and UK Sepsis Trust rules were rarely used. While most GPs had heard of the SIRS criteria, few of the responding GPs had received sepsis training in the past 5 years or had heard of the qSOFA score or UK Sepsis Trust rules. Remarkably, among the GPs unfamiliar with the qSOFA score or UKSTc, most GPs did employ the specific signs and symptoms that make up these criteria to recognize sepsis. When diagnosing sepsis in our fictitious cases, recognition of sepsis varied greatly between GPs. The individual signs of the qSOFA (mental status, systolic blood pressure/capillary refill time and respiratory rate) were parameters GPs most strongly associated with diagnosing sepsis. GP characteristics, like recent in-service sepsis education and use of the ABCDE, did not increase the likelihood of ‘correctly’ identifying cases as sepsis.

### Comparison with existing literature

Research on sepsis in primary care is scarce. To the best of our knowledge, there is only one study that provides insight into the current clinical decision-making process of GPs assessing patients with a potential sepsis. This study, a cross-sectional questionnaire among 160 Dutch GPs, found that general appearance and gut feeling were considered most important when diagnosing a potential sepsis, closely followed by patient history and physical examination. The individual signs of the qSOFA (altered mental status, systolic blood pressure <100 mmHg and respiratory rate ≥22/minute) were considered the three most important aspects of physical examination in this study (15). This study confirms the fact that most GPs use gut feeling when assessing a patient with a potential sepsis. Furthermore, when diagnosing sepsis, the individual signs of the qSOFA were stronger independent predictors than general appearance or aspects of patient history, like recent chemotherapy. Similar to the aforementioned study (15), our results indicate that the qSOFA is in line with the clinical reasoning of GPs. Aside from providing insight into the current decision-making process of GPs when assessing a patient with a potential sepsis, this manuscript also gauged the knowledge and use of various clinical scoring tools.

### Strengths and limitations

This is one of the few studies to provide insight in the current decision-making process of GPs when assessing a patient with a potential sepsis. The fictitious cases in this survey, developed with input from internists specialized in acute medicine, emergency physicians and GPs focussed on research in General Practice Medicine, are designed to gauge the impact of various parameters on the likelihood of sepsis as diagnosis. This design enables to objectively identify parameters that are important for the individual GPs’ diagnosis.

Our study has several limitations: first of all, the response rate was rather low, which may have resulted in selection bias. To maximize response rate, GPs were not only reached out to by mailing lists and a digital newsletter, but also with use of social media (e.g. a nation-wide WhatsApp group). Even though this form of reaching out to GPs resulted in a response rate of only 8.9%, compared with the 20% of the previous questionnaire study in the Netherlands (15), additional analysis (Table 1) shows that relevant characteristics of the responding GPs are similar to the source population. We therefore do not expect this to have influenced our study results. Second, in our cases all vital parameters were given, while, in reality, vital sign measurements like respiratory rate and capillary refill time are frequently not measured (24). Our study shows that these parameters are of great value to GPs when diagnosing sepsis. In line with previous research (16,17), these results indicate that GPs should be encouraged to obtain a full set of vital signs when assessing a patient with a suspected infection.

### Implications for research and/or practice

As shown in our study, most GPs are not familiar with the qSOFA, the recommended screening tool for potentially septic patients outside the ICU according to the new consensus definitions, or the UKSTc, a screening and action tool in line with the most recent guidelines, specifically designed for GPs. Moreover, when asked to determine the likelihood of sepsis in the fictitious cases, answers varied greatly between GPs, with every case being marked as (very) likely being sepsis by one GP and as (very) unlikely being sepsis by another.

Since most patients with sepsis initially contact a GP, this contact is usually the first assessment of a potentially septic patient, and therefore crucial for early recognition and initiation of treatment (14,15). However, due to a lack of information and patients presenting in an early stage of their disease, recognition of sepsis in primary care is poor. In one study of patients admitted to the ICU with a community-acquired sepsis, their GP did not suspect an infection in 43% of cases. This was associated with a significantly higher in-hospital mortality rate (14). Research in pre-hospital settings among Emergency Medical Services (EMS) has shown that the use of screening tools can improve pre-hospital sepsis recognition (12,18). Standardizing recognition of sepsis in primary care, by educating GPs in the use of available screening tools, like the qSOFA or the UK Sepsis Trust rules, may therefore reduce variation between GPs and lower sepsis-associated morbidity and mortality.

Remarkably, despite the fact that most GPs reported to use gut feeling to diagnose sepsis and few had heard of the qSOFA score or UK Sepsis Trust rules, factors associated with recognition of sepsis clearly overlap with these sepsis recognition tools. This may be explained by the fact that clinical diagnostic criteria are based on likelihood from clinical observations, with a combination of findings being associated with an increased likelihood of the presence, or morbidity or mortality due to a certain illness. The GP’s gut feeling is influenced by the GP’s sense of likelihood based on previous experience and knowledge, complemented by a complex interpretation of the patient in clinical context. As both are based on likelihood by clinical observations, the overlap of gut feeling with clinical diagnostic criteria is demonstrated by the fact that GPs diagnose sepsis based on specific factors that are most in line with the qSOFA score, even though they were not familiar with the qSOFA. Gut feeling is an important factor in the diagnostic process of GPs, in sepsis as well as other illnesses. It is, however, hard to objectify and therefore difficult to train (25,26). In contrast, use of screening tools can be trained and could reinforce gut feeling and facilitate decision-making. Thus, gut feeling, which is hard to train, is the most important factor for recognition of sepsis by GPs. Given the overlap between factors associated by the GP’s diagnosis of sepsis and screening tools like the qSOFA and UK Sepsis Thrust rules, educating junior GPs in the use of sepsis screening tools seems a feasible approach to improve sepsis recognition in primary care.

## Conclusions

Even though various screening tools exist to improve sepsis recognition, most GPs use gut feeling to diagnose sepsis. GPs are frequently not familiar with the ‘sepsis-criteria’ used in hospital settings and recognition of sepsis varied greatly between GPs. Nonetheless, GPs most strongly associated the individual signs of the qSOFA with sepsis as diagnosis in the test cases. Together, although GPs report to mostly rely on gut feeling to diagnose sepsis, clinical reasoning is based on specific factors that are most in line with the qSOFA score. In order to improve sepsis recognition in primary care, GPs should be educated in the use of available screening tools, like the qSOFA or the UK Sepsis Trust rules.

## Supplementary Material

cmab020_suppl_Supplementary_Material

## Data Availability

The datasets used and/or analysed during the current study are available from the corresponding author on reasonable request.

## Declaration

Funding: rewards for completing the survey were funded by the emergency department of the University Medical Center Groningen. This study did not receive any further funding.

Ethical approval: as GPs volunteered to participate in a study consisting of a single short questionnaire, the study did not fall under the Medical Research Involving Human Subjects Act (WMO).
