# Supplementary material for: Use of sepsis-related diagnostic criteria in primary care: a survey among general practitioners
Source: Fam Pract. 2021 Mar 23;38(5):617–22. doi: 10.1093/fampra/cmab020 (PMC8527837; doi:10.1093/fampra/cmab020)
Supplement: cmab020_suppl_Supplementary_Material [file cmab020_suppl_supplementary_material.doc]

**Supplemental data**

Table S1. Parameters represented in the cases

| **Parameter** | | **Case 1** | **Case 2** | **Case 3** | **Case 4** | **Case 5** | **Case 6** |
| --- | --- | --- | --- | --- | --- | --- | --- |
|  | Sex, age | Female, 70 y | Female, 28 y | Female, 48 y | Male, 66 y | Male, 63 y | Female, 55 y |
|  | History | Cough, fever, chills, restless for a few hours. Worried family, deterioration. | Renal transplantation. Cough, fever, chills for 3 days. Worried family. | Diabetes mellitus, breast cancer. Worried family, Dysuria for 3 days, fever for 1 day. Bed bound. | COPD. Cough and dyspnea, since yesterday unfit, poor oral intake. Opens door himself. | Fever, near-collapse, unfit for a week, no dyspnea, cough or dysuria. Door opened by home care. | Hypertension, breast cancer. Fever, unfit, no dyspnea, cough, dysuria or skin changes |
|  | General appearance | Exhausted | Tired,  unfit | n/a | Tired,  dyspnea | Near-collapse | Slightly tired,  appearing not ill. |
|  | Systolic blood pressure [mmHg] | 90 | 100 | 105 | 110 | 95 | 110 (160*) |
|  | Pulse [/min] | 95 | 80 | 120 | 100 | 105 | 110 |
|  | Respiratory frequency [/min] | 30 | 18 | 20 | 20 | 25 | 20 |
|  | Oxygen saturation [%] | 90 | 96 | 96 | 90 | 93 | 94 |
|  | Diuresis | n/a | n/a | no | no | yes | n/a |
|  | Mental status [EMV] | 3-6-5, confused | 4-6-5 | 4-6-5 | 4-6-5 | 4-6-5 | 4-6-5 |
|  | Skin | greyish | pale | normal | greyish | greyish and localized erythema of lower leg | normal |
|  | CRT [sec] | <2sec | <2sec | 4 | <2sec | 4 | <2sec |
|  | Recent chemotherapy | n/a | n/a | yes | n/a | n/a | yes |
|  | Body temperature | 39°C | 39°C | 37.5°C | 38.5°C | 37.8°C | 39.5°C |
|  | qSOFA | 3 | 1 | 1 | 0 | 2 | 0 |
|  | SIRS | 3 | 1 | 1 | 2 | 2 | 2 |
|  | UK Sepsis Trust | 5 | 0 | 2 | 3 | 2 | 2 |
|  | Diuresis: urine production (yes/no) in preceding 18 hours, EMV: Eye Motor Verbal (Glasgow coma scale), CRT: capillary refill time, recent chemotherapy: in preceding 6 weeks, * pre-existent systolic blood pressure was provided in case. | | | | | | |


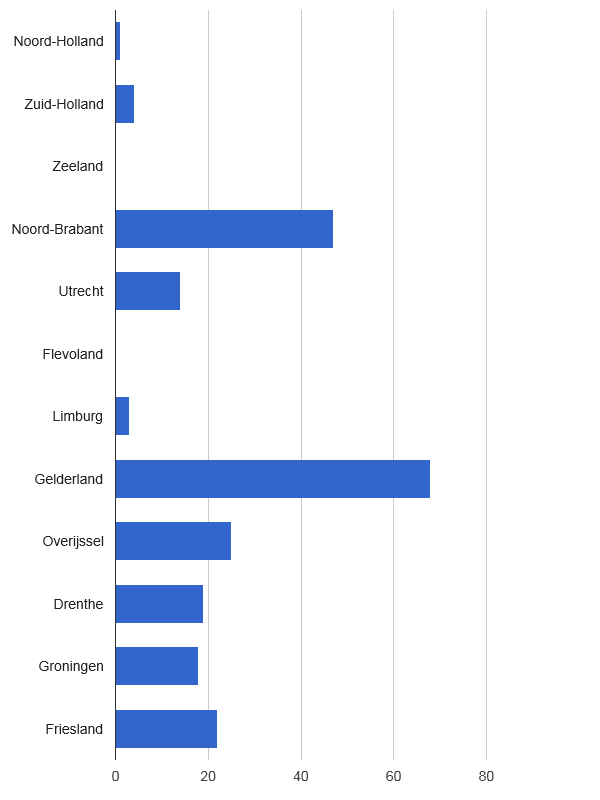


**Figure S1. Location of practice**
